# Lycopene Supplement and Blood Pressure: An Updated Meta-Analysis of Intervention Trials

**DOI:** 10.3390/nu5093696

**Published:** 2013-09-18

**Authors:** Xinli Li, Jiuhong Xu

**Affiliations:** 1 School of Public Health, Medical College of Soochow University, Suzhou 215123, China; 2 Department of Radiotherapy, the First Affiliated Hospital of Soochow University, Suzhou 215006, China; E-Mail: xujiuhong@suda.edu.cn

**Keywords:** lycopene, blood pressure, intervention trials, meta-analysis

## Abstract

Epidemiological studies suggested that lycopene supplement could decrease blood pressure, but the results were conflicting. We conducted an updated meta-analysis by screening PubMed databases, and calculated the combined effect size using a random effect model. In addition, subgroup analysis stratified by baseline blood pressure, lycopene dosage, duration, study location and the funding support of the paper was also conducted. Six studies met our inclusion criteria, and the pooled analysis demonstrated a significant reduction of systolic blood pressure (SBP) (mean SBP = −4.953 [−8.820, −1.086], *p* = 0.012) with obvious heterogeneity (*p* = 0.034, *I*^2^ = 58.5%). Subgroup analysis results showed that higher dosage of lycopene supplement (>12 mg/day) could lower SBP more significantly, especially for participants with baseline SBP >120 mmHg, or Asians, while lycopene intervention had no statistical effect on diastolic blood pressure (DBP) (mean DBP = −3.809 [−8.177, 0.560], *p* = 0.087), and obvious heterogeneity was also observed (*p* = 0.074, *I*^2^ = 53.1%). Our present study suggests that lycopene supplement >12 mg/day might effectively decrease SBP, particularly among Asians or population with higher baseline SBP.

## 1. Introduction

Essential hypertension (EHT), one of the most prevalent chronic diseases, affects nearly a billion people all over the world. It is also a risk factor of cardiovascular morbidity and mortality [1]. Experimental studies have provided strong evidence that oxidative stress, inflammatory processes, endothelial dysfunction and subsequent vascular remodeling have a tight relationship with the pathogenesis of hypertension, especially the role of oxidative stress has been testified by both animal models and human-based studies [2,3]. Oxidative stress could inactivate nitric oxide, impairing endothelium-dependent vasodilatation [4], which suggested that inhibition of oxidative stress might be one effective method controlling blood pressure (BP).

Considering the uncomfortable side effects of antihypertensive drugs and the fact that many hypertensive patients need more than two kinds of drugs per day, alternative and complementary treatment for BP control has been suggested [5,6], such as lifestyle modifications, especially dietary intervention [7,8]. Increasing evidence indicates that dietary consumption of fruits and vegetables decrease BP, which is often ascribed to the role of natural antioxidants—such as lycopene—in improving vascular function [9].

Lycopene, one of the most powerful antioxidants and free radical quenchers, has received attention for its pivotal role in inhibiting oxidative stress, improving vascular function, and preventing cardiovascular disease in humans [10,11,12,13]. However, intervention trials investigating the role of lycopene supplementation or lycopene-containing foods in regulating BP had deduced conflicting results. Several studies demonstrated that at least four weeks of daily oral supplementation with tomato extract or tomato juice significantly decreased BP [9,13,14,15,16], while others showed no relation [17] or no obvious association [18,19]. Paterson *et al*. even found that lycopene (4.5 mg/day, 4 weeks) could elevate BP [20]. One meta-analysis investigating the effect of lycopene on BP had been conducted by Ried *et al*. in 2011 [21], which concluded that lycopene treatment could effectively decrease SBP, but had no statistical effect on DBP. Ried’s meta-analysis only contained four studies, among which included one two-stage, cross-over trial [16] without wash-out period during the intervention, and one self-controlled intervention trial [13]. In regards to the two-stage, cross-over trial [16], Ried *et al*. extracted the combined BP value before and after intervention to conduct the final meta-analysis. As the half life of serum lycopene is about 14 days, the active metabolites and their varying tissue levels may be of importance [22,23,24,25], and the wash-out period may alleviate the effect of lycopene during the treatment of placebo, thus, we think that only the data of BP in stage 1 could be extracted. While about the self-controlled intervention trial [13], Ried *et al*. extracted the baseline BP value as the BP value before lycopene treatment, which we also think is improper. In view of the above fact, and the two published papers since 2011 [14,26], we updated the meta-analysis to establish the current evidence concerning the relationship between lycopene and BP.

## 2. Methods

### 2.1. Search Strategy

PubMed databases was screened using the following search terms (lycopene OR tomato) AND (“blood pressure”). All the intervention studies investigating the effect of lycopene or lycopene-containing products on blood pressure through 2012 had been collected. We restricted our search to “Humans” and that written in English. Reference lists of included articles, reviews and Pubmed option “Related Articles” were also searched for additionally relevant papers. Only studies with full text were included; abstracts or unpublished studies were excluded.

### 2.2. Study Selection

Studies matching the following criteria were included in our final meta-analysis: (1) intervention study; (2) research factors were lycopene and blood pressure; (3) association of lycopene supplement and blood pressure change was evaluated, namely providing the net changes of the BP and their corresponding SDs or available data to calculate these values; (4) characteristics of study were provided, such as intervention dosage of lycopene, intervention duration, BP value at baseline, or before and after intervention; (5) subjects of all ages were accepted.

### 2.3. Data Extraction of Studies

Data were extracted by X-L LI and J-H X independently according to guidelines published by the Cochrane Collaboration [27] and Stroup DF *et al*. [28], and the disagreement was discussed. The characteristics of the studies were extracted, such as study design, samples size, daily dosage of lycopene, duration of intervention, BP value at baseline, before and after treatment with lycopene, characteristics of the participants, such as health status, gender and age were also collected. If the research had investigated different dosages of lycopene with one control arm, only the data of the higher intervention dose was extracted. In regards to the placebo-controlled two-group, two-stage, cross-over trial, if the design was without washout period, we only extracted the data of stage one, namely the lycopene and placebo interventions in stage one were regarded for parallel study.

### 2.4. Quality Assessment of Studies

The methodological quality of each included trial was assessed by X-L LI and J-H XU independently according to the Jadad scale [29]. Key components of study designs, including whether randomization, blinding, the rate of loss to the follow-up, and the compliance of the participant were employed.

### 2.5. Statistical Methods

The weighted mean difference (WMD) of BP value was used to assess the association between lycopene supplement and BP change. Standard deviations (SD) of the mean difference were calculated using the formula: square root [(SDtime1)2 + (SDtime2)2 − 2*R* × SDtime1 × SDtime2], and a correlation coefficient *R* = 0.5 according to the guidelines of the Cochrane Collaboration [27].

Heterogeneity assumption across studies was tested by a chi-square-based Q-test. A *p* value of more than 0.10 indicated a lack of heterogeneity, *I*^2^ statistic was also calculated. If the heterogeneity test is statistically significant, the pooled estimation of the WMD was calculated by the random effects model (Mantel-Haenszel method), otherwise, the fixed effects model (DerSimonian and Laird method) was employed [30].

The effect of various study characteristics on pooled outcomes, including baseline SBP and DBP, dosage of lycopene, length of intervention, geographic regions, or whether they were supported by funds were examined in subgroup analyses.

Sensitivity analysis was performed using both fixed- and random-effect models to evaluate the effect of single study on the overall outcomes by omitting one study each time.

Potential publication bias was assessed by Begg’s funnel plots test (*p* < 0.10) [31]. If there was an asymmetric plot, it would suggest a possible publication bias.

All analyses were performed using STATA version 11.0 (Stata Corp). A *p* value < 0.05 was considered significant, except where specified.

## 3. Results

### 3.1. Literature Selection

**Figure 1:**
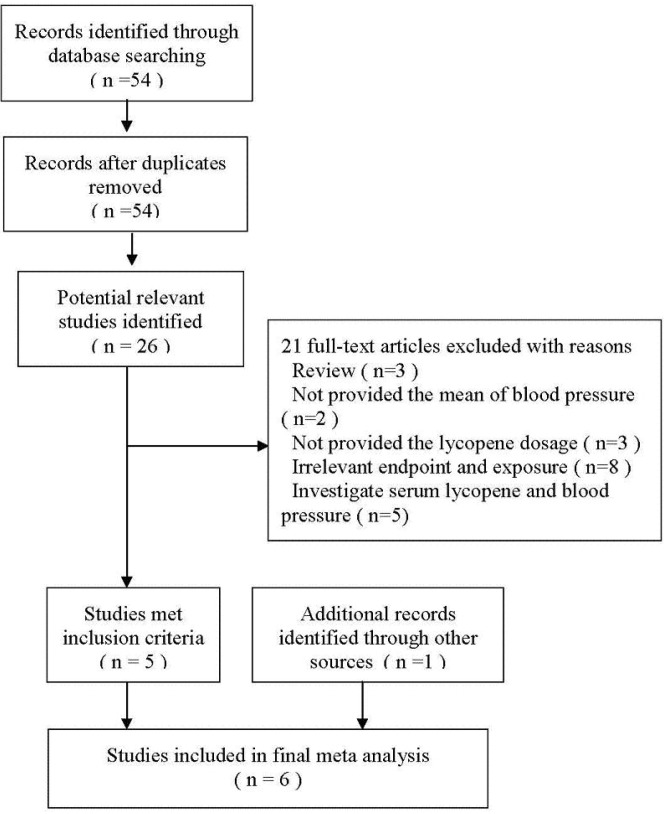
Flow diagram of paper search.

As shown in Figure 1, 54 publications from the Pubmed database were collected using the search terms (lycopene OR tomato) AND (“blood pressure”), and most studies were excluded because they were not intervention trials or they had irrelevant exposure or end-point. By reviewing the full-text of the 26 potentially relevant articles, five studies met our inclusion criteria [13,14,15,16,26], and one new study was added [20] after reviewing the reference lists of retrieved articles. Twenty-one studies were excluded for the following causes: three studies were systematic reviews [21,26,27]; two studies could not provide the mean BP value [19,32]; three did not provide the lycopene dosage [31,33,34]; eight had irrelevant endpoint and exposure [18,28,30,35,36,37,38,39]; and five investigated the association between serum lycopene and blood pressure [9,17,40,41,42].

Among the six studies included in our final analysis, two studies had investigated the effect of lycopene intervention on BP with two levels of lycopene treatment respectively [14,26], and only data of higher intervention dosages were extracted, thus six groups of data sets were extracted.

### 3.2. Characteristics of the Included Studies

Characteristics of the included studies were displayed in Table 1. Three studies were conducted in Asia [13,14,16], two in Europe [20,26], and one in Oceania [15]. Four studies were paired with a placebo-controlled group [13,14,15,16], and two with a lycopene-free diet controlled group [20,26]. Two studies used a lycopene-containing diet as test intervention [20,26], others used Lyc-O-Mato tomato extract capsules. The intervention dosages of lycopene ranged between 4.5 and 15 mg/day, with a mean dosage of 12.4 mg/day. The treatment periods ranged between four and 16 weeks, with a mean duration of 8.3 weeks. Two studies selected hypertensions [13,16], and one selected prehypertensions as subjects [15], and Kim *et al*. [14] only selected middle age males as subjects, the other two [20,26] were healthy adults.

### 3.3. Quality Assessment of Selected Studies

Quality assessment of all studies included in our final analysis was conducted (Table 2). All studies were blinding to participants in trials. There were two non-randomized trials [13,16], and most of the trials did not report allocation concealment, but were practical in meta-analysis methodology.

The drop-out rates ranged between 0 and 8.9%, and the compliance was satisfactory. All trial subjects were suggested to maintain their usual lifestyle and dietary habits, no other dietary supplements were allowed, and dietary assessment was also conducted. Meanwhile, five studies [13,14,16,20,26] measured the blood level of lycopene at baseline, pre and post treatment to assess the compliance or by counting the remaining capsules [13,14,15,16] and the return of unused products [20]. Four trials reported receipt of food industry funding [14,15,20,26], and two trials did not report the funding sources [13,16].

### 3.4. Meta-Analyses Results

#### 3.4.1. Effect of Lycopene Supplement on Blood Pressure

**Figure 2:**
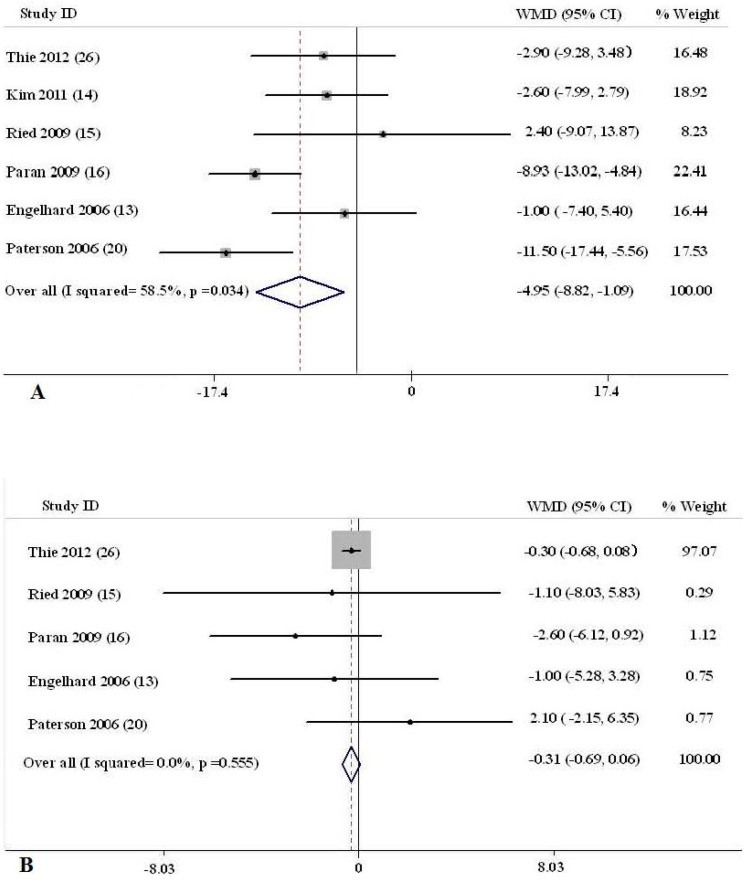
Meta-analysis of the effect of lycopene on blood pressure in the random effect model. WMD, weighted mean difference. (**A**): Systolic blood pressure; (**B**): Diastolic blood pressure.

The net changes and the corresponding 95% CIs for SBP in each trial and overall are presented in Figure 2A. Compared with no intervention (control), lycopene supplement was associated with an average net change in SBP ranging from −11.50 to 2.40 mmHg. SBP was decreased in response to lycopene intervention in five of the six trials, among which two trials had statistical reduction of SBP. The overall pooled estimate of the lycopene treatment on SBP was −4.953 mmHg (95% CI, −8.820, −1.086, *p* = 0.012). Tests for heterogeneity showed significant differences across studies (*p* = 0.034, *I*^2^ = 58.5%), thus the random effects model was employed.

**Table 1:** Characteristics of included studies.

Study, year (reference), region	Study design	Source of lycopene/control	Dosage (mg)/day	Duration	Change of BP treatment *vs.* control	Participants, m/f, age	Sample size	Other source of lycopene	Assessment of dietary intake
Thie, 2012 [26], Aberdeen, Scotland	single-blind, RCT	L1: tomato extract capsule (purchased from Holland and Barret) L2: tomato-based foods C: placebo capsule	L1: 10 L2: 10 C: 0	16 weeks	SBP: −3.2 *vs.* −0.3 DBP: −1 *vs.* −0.7	men and women; aged 51 years	L: 68 T: 81 C: 76	no other dietary supplements were allowed	by using seven-day food diaries before and during the run-in period as well as during the intervention
Kim, 2011 [14], Yonsei, Asia	double-blind, RCT	L: tomato extract capsule (Lyc-O-Mato) C: placebo capsule	L1: 6 L2: 15 C: 0	8 weeks	SBP: −3.2 *vs.* −0.6	healthy male, smoker or alcohol-drinker, low intake of fruits and vegetables; male, 33.5–34.8 years	L1:41 L2: 37 C: 38	negligible	by 24-h recall method and semi-quantitative food frequency questionnaire
Ried, 2009 [15], Australia, Oceania	double-blind, RCT, three-group parallel trial	L: tomato extract capsule (Lyc-O-Mato) C: Placebo capsule	L: 15 C: 0	8 weeks	SBP: −2.5 *vs.* −4.9 DBP: −1.6 *vs.* −0.5	prehypertensive adults, with no antihypertensive drugs; 12 m/13 f, 52 ± 12 years	L: 15 C: 10	negligible	by questionnaires and participants’ daily diary entries
Paran, 2009 [16], Israel, Asia	double-blind, placebo controlled, two-group crossover trial	L: tomato extract capsule (Lyc-O-Mato) C: placebo capsule	L: 15 C: 0	6 weeks	SBP: −13.6 *vs.* −2.1 DBP: −4.2 *vs.* −2.1	mild hypertensives, with one or two antihypertensive drugs; 26 m/24 f, 56 ± 10 years	L: 50 C: 50	no other dietary supplements were allowed	by dietary query
Engelhard, 2006 [13], Israel, Asia	single-blind, placebo controlled trial	L: tomato extract capsule (Lyc-O-Mato) C: placebo capsule	L: 15 C: 0	8 weeks	SBP: −9.98 *vs.* −1.05 DBP: −4.06 *vs.* −1.46	mild hypertensives, non-smokers with no antihypertensive drugs, 18 m/13 f, 52 ± 21 years	L: 31 C: 31	no other dietary supplements were allowed	by dietary questionnaire
Paterson, 2006, [20], U.K, Europe	single-blind, RCT	L: carotenoid-rich canned soups C: carotenoid-poor canned soups	L: 4.5 C: 0	4 weeks	SBP: 1 *vs.* 2 DBP: 1 *vs.* 2	healthy adult, 12 m/24 f, 43.5 ± 23.5 years	L: 36 C: 36	included	by a three-day estimated diet diary

RCT: randomized controlled trial.

**Table 2:** Quality assessment of included studies.

Study ID	Randomization	Allocation concealment	Blinding	Loss to follow-up	Dietary advice	Compliance	Funding source
Thie, 2012, Scotland [26]	randomized		single-blind	22/247	control group was restricted	assessed by measuring serum lycopene concentrations and by analyzing a weekly checklist of tomato-based foods consumed.	funding from the Scottish Government (RESAS).
Kim, 2011, Yonsei [14]	randomized		double-blind	10/126	maintain their usual lifestyle and dietary habits	assessed using pill counting, food records, and measurement of plasma lycopene levels	National Research Foundation, Korea Health 21 R & D Projects
Ried, 2009 Australia [15]	permuted block randomization using the SAS 9.1 software package	sequentially numbered containers	double-blind	3/39	maintain their usual diet and physical activity	assessed using participants daily diary entries	RACGP 2006 Pfizer Cardiovascular Research Grant, Australian Government Primary Health Care Research Evaluation Development (PHCRED) Program
Paran, 2009, Israel [16]	non-randomized	?	double-blind	0/50	no other dietary supplements were allowed and to keep their usual dietary and exercise habits	verified by counting the remaining capsules and by reinforcement at each visit	No funding source provided
Engelhard, 2006, Israel [13]	non-randomized	?	single-blind	3/34	no other dietary supplements were allowed and to keep their usual dietary habits	by counting the remaining capsules and by reinforcement at each visit	no funding source provided
Paterson, 2006, UK [20]	block-randomization stratified by age, gender, BMI	?	single-blind	0/36	comprehensive food diaries	assessed by the return of unused products at the end of each intervention period	Unilever Best foods and the University of Reading Research Endowment Trust Fund

As displayed in Figure 2B, lycopene intervention had no statistically significant effect on decreased DBP compared with the control group, the average net change in DBP ranged from −0.30 to 2.10 mmHg, and the overall pooled estimate of lycopene on DBP was −3.809 mmHg (95% CI, −8.177, 0.560, *p* = 0.087). Tests for heterogeneity also showed significant differences across studies (*p* = 0.074, *I*^2^ = 53.1%), thus the random effects model was employed to calculate the pooled outcome.

#### 3.4.2. Results of Subgroup Analyses

Subgroup analyses were conducted to investigate the effect of the baseline BP value, dosage of lycopene, duration of intervention and geographic regions on the pooled results. As presented in Table 3, significant reduction of SBP was observed if the study was conducted in Asia [mean SBP = −7.661 mmHg (−12.480–−2.842), *p* = 0.002], or participants had higher baseline SBP (SBP ≥ 120 mmHg) [mean SBP = −8.034 (−12.411–−3.656), *p* = 0.000], higher intervention dosage of lycopene (>12 mg/day) [mean SBP = −6.350 (−11.342–−1.358), *p* = 0.013]. Duration of intervention and support of funding had no significant effect on SBP. As shown in Table 4, subgroup analysis had not deduced any statistical effect on DBP.

**Table 3:** Results of stratified analyses of the blood pressure, A: Systolic blood pressure.

Group	Total data included	WMD (95% CI)	*p*	*p* for heterogeneity	*I*^2^, %
**All**	**6**	**−4.953 (−8.820, −1.086)**	0.012	0.034	58.5
**Baseline of SBP**					
<120 mmHg	3	−1.441 (−5.320, 2.439)	0.467	0.731	0.0
>120 mmHg	3	**−8.034 (−12.411, −3.656)**	0.000	0.139	49.4
**Dosage of lycopene**					
<12 mg/day	2	−1.953 (−6.473, 2.568)	0.397	0.680	0.0
>12 mg/day	4	**−6.350 (−11.342, −1.358)**	0.013	0.042	63.4
**Duration of intervention**					
>8 weeks	4	−4.324 (−8.753, 0.105)	0.056	0.100	52.1
<8 weeks	2	−6.320 (−16.609, 3.969)	0.229	0.018	82.0
**Location**					
Asia	3	**−7.661 (−12.480, −2.842)**	0.002	0.069	62.7
Other regions	3	−1.368 (−5.573, 2.838)	0.524	0.723	0.0
**Funding**					
Support by funding	4	−4.481 (−9.821, 0.859)	0.100	0.061	59.3
No-support by funding	2	−5.363 (−13.095, 2.369)	0.174	0.041	76.1

**Table 4:** Results of stratified analyses of the blood pressure, B: Diastolic blood pressure.

Group	Total data included	WMD (95% CI)	*p*	*p* for heterogeneity	*I*^2^, %
**All**	5	−0.315 (−0.687, 0.057)	0.097	0.555	0
**Baseline of DBP**					
<80 mmHg	3	−0.308 (−0.683, 0.068)	0.108	0.927	0.0
>80 mmHg	2	−0.408 (−5.003, 4.188)	0.862	0.095	64.1
**Dosage of lycopene**					
<12 mg/day	2	−0.305 (−0.681, 0.071)	0.111	0.750	0.0
>12 mg/day	3	−0.629 (−3.746, 2.489)	0.693	0.247	28.6
**Duration of intervention**					
>8 weeks	3	−0.328 (−0.703, 0.046)	0.086	0.434	0.0
<8 weeks	2	0.562 (−2.476, 3.600)	0.717	0.314	1.4
**Location**					
Asia	2	−0.408 (−5.003, 4.188)	0.862	0.095	64.1
Other regions	3	−0.308 (−0.683, 0.068)	0.108	0.927	0.0
**Funding**					
Support by funding	3	−0.284 (−0.659, 0.092)	0.139	0.530	0
No-support by funding	2	−1.956 (−4.674, 0.763)	0.159	0.572	0

#### 3.4.3. Sensitivity Analyses

In the sensitivity analyses, omitting the trials by Engelhard *et al*. [13] or Paran *et al*. [16] resulted in an insignificant reduction of SBP by using random-effect model, while analysis using fixed-effect model showed that no trials had substantial influence on the pooled analysis, and the results ranged from −4.128 (95% CI: −7.024, −1.233) to −5.738 (95% CI: −9.945, −1.530) mmHg. From the results of DBP, none of trials seemed to substantially influence the effect of lycopene.

#### 3.4.4. Publication Bias

**Figure 3:**
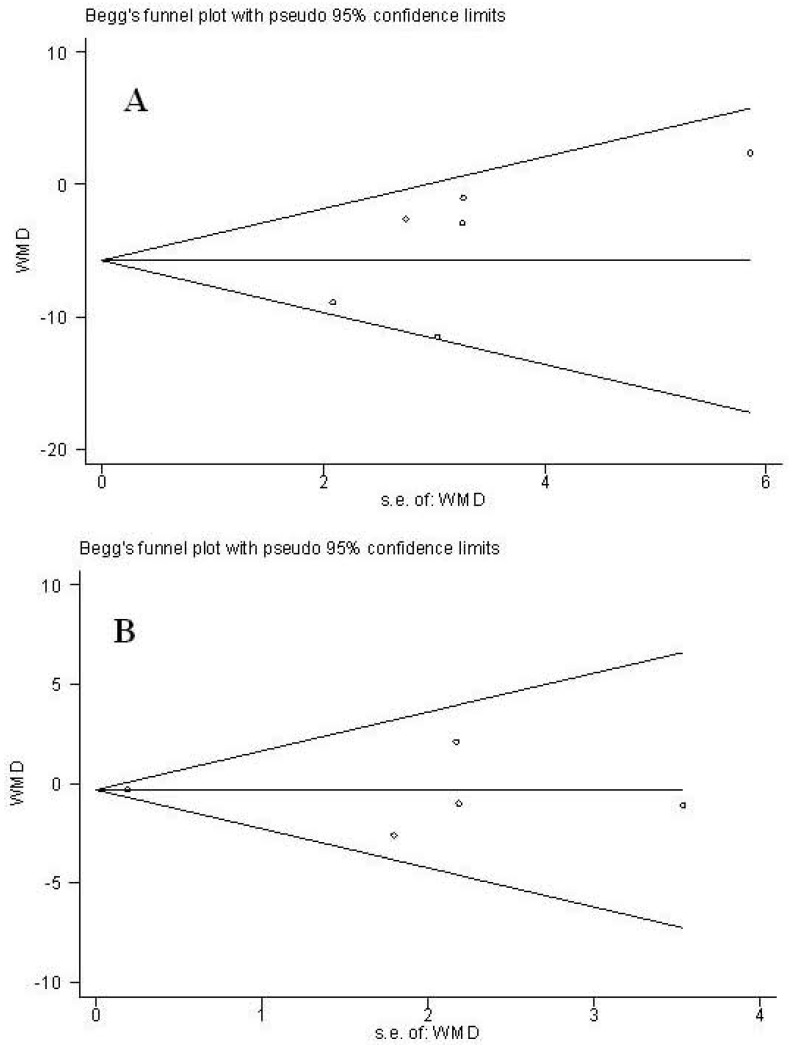
Funnel plot analysis to detect publication bias. (**A**): Systolic blood pressure; (**B**): Diastolic blood pressure.

As displayed in Figure 3, results of Funnel plots and Egger’s test of trials showed no publication bias (*p* = 0.192 and 0.751 for SBP and DBP, respectively).

## 4. Discussion

Our present meta-analysis about the effect of lycopene on BP demonstrated that lycopene supplement had a beneficial effect on SBP, as supported by significantly decreased SBP (mean SBP = −4.953 mmHg [95% CI, −8.820, −1.086, *p* = 0.012]), and had no statistical effect on DBP (mean DBP = −3.809 mmHg [95% CI, −8.177, 0.560, *p* = 0.087]). Our results were consistent with the previous meta-analysis [30].

Obvious heterogeneity was observed across our study, which could be explained by the inconsistency in the participants’ collection; healthy adults, prehypertensive patients and hypertensive patients with or without antihypertensive drugs were collected. Meanwhile, studies involved in our meta-analysis were conducted in different geographic regions, and the participants might share different genetic background, lifestyle, HT incidence and sensitivity to lycopene.

Results from subgroup analyses indicated that lycopene could effectively lower SBP of prehypertensive or hypertensive subjects, and a higher dosage of lycopene appeared to be more effective in reducing SBP than a low dosage. Furthermore, the beneficial effect of lycopene on SBP was more obvious among Asians than other regional populations; this might be ascribed to the different genetic background and lifestyle. For example, consumption of vegetable foods, which contains plenty of vitamin and phytochemicals, was higher in Asian people than other regional populations.

For the participants of all the included trials, those who take regular supplements, such as antioxidant, vitamin, or mineral supplements, or any nutrients that known to affect any variable determined were excluded according to their daily diet and dietary habits. Meanwhile, no other dietary supplements were allowed and they were asked to keep their usual dietary and exercise habits during the study, so the change of SBP was unlikely to be attributed to weight lowering, major dietary changes, or enhanced physical activity. The determination of blood lycopene level and the dietary assessment during the intervention also supported the role of lycopene in regulating SBP. In fact, during the intervention, the BMI or weight of all participants had no obvious change. Thus, our results provided certain evidence that lycopene might play a certain role in lowering SBP, which suggested the possibility of controlling SBP by lycopene supplements, especially among prehypertensive or hypertensive populations. Actually, the role of lycopene in lowering SBP might be attribute to its role as an antioxidant and free radical quencher, which could inhibit oxidative stress, indirectly stimulate production of nitric oxide in the endothelium [34], and improve vascular function.

About the individual studies, there lied some discrepancy: (1) Subjects collection. Kim *et al*. [14] only selected middle age male as subjects, those population shared different incidences of HT compared with females. Engelhard *et al*. [13] and Paran *et al*. [16] collected hypertensive participants as subjects; (2) The frequency of BP assessments was also different. In Paran’s study, BP is measured every three weeks [16], Engelhard’s is every two weeks [13], and Paterson’s only determined BP before and after the intervention [20]; (3) Study design was different. Two studies included washout period [15,20], and two studies included run-in period [13,20]. The inclusion of washout and run-in periods could authentically reflect the role of lycopene and exclude the interference of other research factors.

Our results from the sensitivity analysis showed that the pooled result was not affected by using a fixed effect model. When omitting the trial of Engelhard *et al*. [13] and Paran *et al*. [16], the size of the pooled effect was overturned by using a random effect model. The above results implied that heterogeneity is the main factor that affected our result from sensitivity analysis, thus more trials with large sample sizes are required in the future to eliminate the effect of heterogeneity and to confirm our results. Furthermore, the study of Engelhard *et al*. [13] and Paran *et al*. [16] selected hypertensive patients as subjects; the change in pooled results after omitting these above two studies each time suggested that lycopene treatment was more efficient for hypertensive populations than normal population. In other words, lycopene could decrease higher BP, but had no effect on normal BP.

Except for the variables included in our study, other factors should also be considered. In our present meta-analysis, the highest dose of lycopene administered was 15 mg/day; the duration of intervention was over a 4–16 week period. As Reich’s study showed that the dose of lycopene of up to 200 mg daily long-term appeared to cause minimal side effects [35], and treatment of prehypertension may forestall progression to hypertension and decrease risk of cardiovascular morbidity and mortality later in life [7], this has important clinical significance, thus further research with higher dosage of lycopene and a longer intervention duration is needed to investigate the effect of lycopene on BP, especially among prehypertensive subjects.

Several limitations in our current study should be addressed. First, only a small number of trials (*n* = 6), with a relatively small sample size, have been included in our study. We may not have been able to detect any differences in blood pressure smaller than 5 mmHg in SBP or 3 mmHg in DBP between groups. Second, due to the small sample size, we failed to determine the role of lycopene in regulating BP stratified by the types of lycopene products, and to assess the dose dependency between the increase of lycopene dosage and the decrease of SBP or DBP. In light of the strong inverse relationship between lycopene and SBP, we can deduce that the observed decrease in SBP is the result of lycopene supplementation. Third, obvious heterogeneity was observed, and the random model was employed. Fourth, the study design of the trials in our meta-analyses is inconsistent, which included parallel and cross-over trials, as well as trials with repeated measure design. Whether the inclusion of an initial run-in period and the washout period during the intervention would affect the result is debatable. Because of the limited trials, we could not further analyze the effect of study design on the pooled results. Although the methodology is practical to combine the data from different study designs, more high quality trials are needed to confirm our findings.

There are several advantages to our study. Although only six studies were involved in our meta-analysis, it could provide relatively more statistical power and reliable estimates than individual studies to detect the association between lycopene treatment and BP. The original studies included in our final meta-analysis were all prospective, clinical intervention studies, which greatly reduced the likelihood of recall bias and selection bias, especially as RCT provided much stronger support for a causal association than observational studies.

In comparison with previous meta-analysis, although there were no new findings in our present study, our results, with a relatively larger sample, at least re-testified that the lycopene supplement had a beneficial effect on SBP and provided subgroup analysis results. Meanwhile, our results have important public health implications. As a common disease among adults, HT is now a burden for both individuals and society. In view of the side-effects of antihypertensive drugs, dietary intervention is now more popular. Our findings about the role of lycopene in lowering SBP are therefore important and timely.

## 5. Conclusions

In conclusion, our meta-analysis provides evidence of the role of lycopene in lowering SBP; thus, to provide lycopene or tomato extract as effective additions for antihypertensive treatment, longer term studies with a larger number of patients are required in the future.

## Author Contributions Statement

Jiuhong XU and Xinli LI conducted the literature search, determined studies for exclusion and inclusion, extracted data from retrieved studies, performed the meta-analysis, and drafted the manuscript of the methods. Xinli LI recovered the publications, determined studies for exclusion and inclusion, extracted data from retrieved studies and drafted the manuscript. All authors approved the final manuscript.
